# Sputtered AlN Buffer Layer for Low-Loss Crystalline AlN-on-Sapphire Integrated Photonics

**DOI:** 10.1021/acsphotonics.5c02661

**Published:** 2026-02-10

**Authors:** Samuele Brunetta, Samantha Sbarra, Brandon Shuen Yi Loke, Jean-François Carlin, Nicolas Grandjean, Camille-Sophie Brès, Raphaël Butté

**Affiliations:** † Laboratory of Advanced Semiconductors for Photonics and Electronics, 150727 Ecole Polytechnique Fédérale de Lausanne (EPFL), Lausanne CH-1015, Switzerland; ‡ Photonic Systems Laboratory, 27218 Ecole Polytechnique Fédérale de Lausanne (EPFL), Lausanne CH-1015, Switzerland

**Keywords:** aluminum nitride, microring resonator, voids, photonic integrated circuits, metalorganic vapor-phase epitaxy, sputtered buffer layer

## Abstract

In recent years, aluminum nitride (AlN) has emerged as an attractive material for integrated photonics due to its low propagation losses, wide transparency window, and presence of both second- and third-order optical nonlinearities. However, most of the research led on this platform has primarily focused on applications rather than material optimization, although the latter is equally important to ensure its technological maturity. In this work, we show that voids, which are commonly found in crystalline AlN-on-sapphire epilayers, have a detrimental role in related photonic structures, as they can lead to propagation losses exceeding 30 dB cm^–1^ at 1550 nm. Their impact on light propagation is further quantified through finite-difference time-domain simulations that reveal void-related scattering losses are strongly dependent on their size and density in the layer. As a possible solution, we demonstrate that when introducing a thin sputtered AlN buffer layer prior to initiating AlN epitaxial growth, void-free layers are obtained. They exhibit intrinsic quality factors in microring resonators as high as 2.0 × 10^6^, corresponding to propagation losses lower than 0.2 dB cm^–1^ at 1550 nm. These void-free layers are further benchmarked for high-power applications through second-harmonic and supercontinuum generation in dispersion-engineered waveguides. Such layers are highly promising candidates for short-wavelength photonic integrated circuit applications, particularly given the strong potential of AlN for visible photonics. Given that volumetric scattering losses scale as λ^–4^, the platform quality becomes increasingly critical in the visible and ultraviolet range, where our improved layers are expected to deliver enhanced performance.

## Graphical abstract

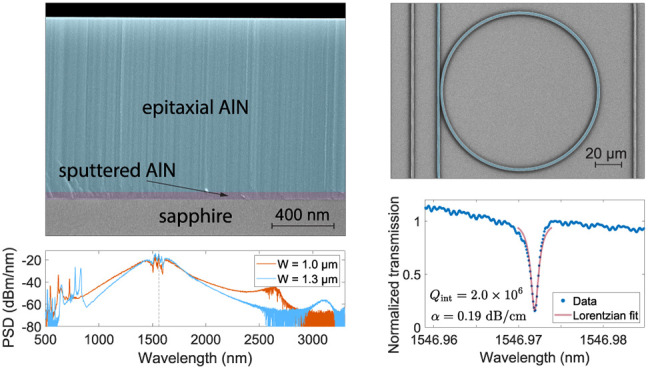

## 1 Introduction

In the past decade, aluminum nitride (AlN) has emerged as an attractive material for linear and nonlinear integrated photonics due to its outstanding properties. Chief among them is its wide transparency window, which ranges from 210 nm to beyond 8 μm,^1,2^ enabling a broad range of photonic applications from the ultraviolet^3^ to the mid-infrared.^2^ In addition to a third-order optical nonlinearity, mediated by the nonlinear refractive index *n*_2_ = 3.5 × 10^–19^ m^2^ W^–1^ of the same order as for competing platforms such as silicon nitride (Si_3_N_4_) and thin-film lithium niobate (TFLN)^4–6^ AlN also possesses an intrinsic second-order susceptibility χ^(2)^ originating from its noncentrosymmetric wurtzite cell structure. These aspects, together with its suitability to handle high optical powers due to its relatively small thermo-optic coefficient (d*n*_op_/d*T* ≃ 2.3 × 10^–5^ K^–1^ at 1550 nm)^7^ and good thermal conductivity (κ = 285 W m^–1^ K^–1^),^8^ make it a sound choice for nonlinear optical applications.^9–12^ Furthermore, AlN photonic integrated circuits (PICs) recently enabled to achieve narrow-line width lasing via self-injection locking (SIL) operation of Fabry–Perot laser diodes in the infrared range,^13^ at 780 nm,^14^ and in the blue,^15^ with potential for applications such as optical atomic clocks. Moreover, owing to its Pockels coefficients (*r*_33_ and *r*_31_) on the order of 1 pm V^–1^, this material platform also supports the fabrication of integrated electro-optical modulators^16–18^ which can be readily combined with SIL-based laser architectures for fast frequency modulation.

Although the first AlN-based PICs were fabricated from sputtered AlN layers on SiO_2_-on-Si substrates,^19^ since 2017 most of AlN PIC studies focused on AlN layers grown on *c*-plane sapphire by metalorganic vapor-phase epitaxy (MOVPE).^20^ The latter show improved material quality enabling the fabrication of microring resonators (MRRs) with intrinsic quality factors (*Q*_int_) up to 3.7 × 10^6^ at 1550 nm (corresponding to propagation losses of 0.1 dB cm^–1^),^21^ against a *Q*_int_ value limited to 8 × 10^5^ for sputtered AlN.^22^ Since then, a wide range of works dealing with nonlinear and quantum optics have been reported^23,24^ but few efforts have been devoted to understanding the mechanisms responsible for optical losses. This research direction could open the door to further advances with this platform, as reported successfully with Si_3_N_4_ ^25–28^ and TFLN.^29^

In particular, due to the heteroepitaxial nature of the growth and the strategies commonly used to produce optoelectronic-grade layers, AlN epilayers grown on sapphire often contain voids.^30^ While such a feature can be beneficial for short-wavelength optoelectronic applications as voids filter dislocations, relieve strain to support the growth of thicker layers,^31,32^ and could facilitate light extraction, they may pose some challenges in the field of integrated photonics. Indeed, voids could act as efficient scattering centers that strongly degrade optical performance, an issue that becomes all the more critical for short wavelength applications, such as chip-scale self-injection locked lasers of use for compact optical atomic clocks, and quantum sensing, since volumetric scattering losses are expected to scale as λ^–4^.^33^

In this work, we investigate the impact of voids in AlN-on-sapphire waveguiding structures. We show that voids, commonly found in MOVPE-grown layers, can lead to propagation losses exceeding 30 dB cm^–1^ at a wavelength of 1550 nm, as measured in both waveguides (WGs) and MRRs. Such level of losses is further supported by finite-difference time-domain (FDTD) simulations, which reveal their strong sensitivity to the size and density of voids. Moreover, simulations indicate that relatively small voids, which have a negligible impact at telecom wavelengths, may play an important role in propagation losses at shorter wavelengths, highlighting voids as a potential limiting factor in current state-of-the art visible AlN photonics. Finally, we experimentally show that “hybrid″ AlN layers, consisting of MOVPE-grown AlN on a thin sputtered AlN buffer on sapphire, being void-free, offer a powerful solution to this problem. With such layers, we achieve integrated waveguiding structures at 1550 nm with low propagation loss <0.2 dB cm^–1^, and we confirm their suitability for nonlinear photonic applications with the demonstration of second-harmonic generation (SHG) and supercontinuum generation (SCG) in dispersion-engineered WGs.

## 2 Results and Discussion

### 2.1 Material Characterization

In this work, we studied three different types of AlN epilayers grown on 2-inch *c*-plane sapphire substrates. Sample A was grown with an Aixtron 200/4 RF-S horizontal reactor, using a full-MOVPE process, which includes a thin AlN nucleation layer grown at lower temperature to facilitate the start of the heteroepitaxial growth process. Sample B is a 1.0 μm-thick commercially available epilayer purchased from DOWA Electronics Materials Co., Ltd. Sample C relies on an in-house hybrid process, which consists in growing the main AlN layer by MOVPE on top of a 50 nm-thick sputter-deposited AlN buffer layer on *c*-plane sapphire, provided by Evatec AG. The main characterization parameters are shown in Table 1, while additional details and data are reported in the Supporting Information (SI) Section S1. The three layers share a comparable value of the root-mean-square (RMS) surface roughness, such as deduced from atomic force microscopy (AFM). However, X-ray diffraction (XRD) data indicate that sample A has both a poorer crystalline quality and a higher degree of off-plane crystal tilt and in-plane twist compared to samples B and C, as shown by the full width at half-maximum (fwhm) of the symmetric (0002) peak and that of the asymmetric (213̅1) peak (Table 1), respectively.

**Table 1:** Material Characterization Data of the Three AlN-on-Sapphire Epilayers Investigated in This Work

	Sample A	Sample B	Sample C
Sample details	Full-MOVPE	Commercially available	Hybrid^a^
AlN thickness^b^ (μm)	1.1	1.0	1.1
RMS surface roughness^c^ (nm)	0.17	0.11	0.09
XRD (0002) peak fwhm	0.151°	0.029°	0.023°
XRD (213̅1) peak fwhm	0.474°	0.410°	0.441°
Void density *N* (μm^–2^)	123 ± 11	41 ± 6	None
Void height *H* (nm)	40–300	20–80	None
Void diameter *d*, deduced from SEM (nm)	7–13	4–8	None
Void diameter *d*, deduced from AFM (nm)	39–53	39–51	None

a MOVPE regrowth on a sputtered buffer layer.

b Measured at the wafer center.

c Over a 2 × 2 μm^2^ area.

In order to further probe the material quality of these samples, we performed cross-section scanning electron microscopy (SEM) imaging on the three epilayers. The SEM images shown in Figure 1a–c reveal that, in addition to vertical striations resulting from the cleavage process, samples A and B both contain vertically elongated air voids within the AlN layer near the interface with the sapphire substrate. No such voids were observed on sample C. This feature is accounted for by the fact that the full MOVPE recipe used for growing sample A was optimized to produce Al-polar crack-free AlN layers, as commonly done to obtain device-grade short-wavelength III-nitride Al-rich optoelectronic devices.^31^ This recipe starts with the deposition of a low-temperature AlN nucleation layer that promotes the Al-polarity of the growing surface, followed by high-temperature AlN growth. The growth parameters during the early stage of the high-temperature growth are tuned to induce three-dimensional (3D) growth, to avoid the appearance of cracks and improve the crystalline quality. Therefore, the voids in sample A are certainly formed during the coalescence of the 3D layer. Unlike sample A, the use of a thin sputter-deposited AlN buffer layer prior to initiating MOVPE growth for sample C did not require any 3D-growth stage to obtain crack-free epilayers, hence leading to void-free layers. Although voids are often used to mitigate crack formation,^32^ the absence of voids in sample C did not significantly increase cracking. Only a few isolated cracks were observed in sample C, similar to sample A. This indicates that cracks in our case are mainly determined by growth parameters and suggests that hybrid AlN epilayers up to 1.3 μm thick may be grown without significant cracking.

**Figure 1:**
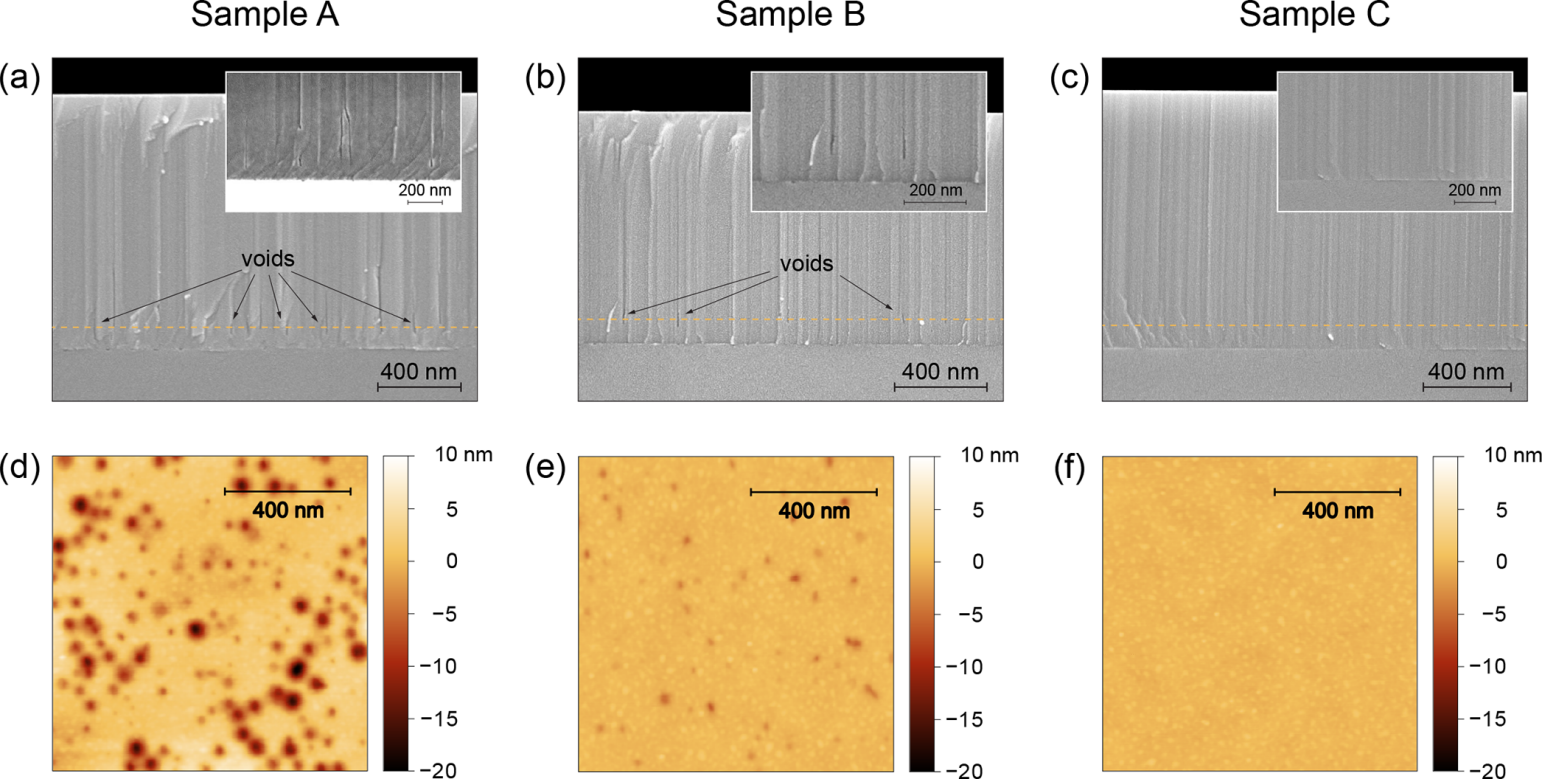
(a–c) Cross-section SEM images of the epilayers A, B and C, respectively, with a high magnification inset for the AlN-sapphire interface region. Samples are coated with a thin metal layer (8–10 nm) for charge-dissipation purposes. (d–f) Surface AFM scans of the epilayers A, B and C, respectively, after thinning them down by ICP dry etching to a thickness of 110 nm (horizontal dashed orange lines in the SEM pictures).

Since voids are generally elongated in the vertical direction, we will refer to their in-plane size, usually in the few tens of nanometer range, as their diameter (*d*), while their vertical size, whose value can exceed a few hundreds of nanometers,^32^ will be referred to as their height (*H*). Multiple cross-section SEM pictures were analyzed, and measurements were extracted from 27 voids in sample A and 11 voids in sample B. The measured *H* values resulted in a spread from 40 to 300 nm for sample A and 20–80 nm for sample B. As for the void diameter, *d* values were found to lie in the range of 7–13 nm and 4–8 nm in samples A and B, respectively. However, these values are likely underestimated due to the use of a thin metal coating for mitigating charge accumulation during imaging and the inherent difficulty of accurately measuring irregularly shaped narrow features in cross-section SEM images.

To determine the areal density of voids *N* in the films, the three AlN layers were etched using inductively coupled plasma (ICP) dry etching down to a thickness of (110 ± 9) nm (indicated by the horizontal orange dashed line in Figure 1a–c), corresponding to the depth where most of the voids are located. AFM images of the etched surfaces are shown in Figure 1d–f, from which we could extract a void density *N* of (123 ± 11) μm^–2^ and (41 ± 6) μm^–2^ in samples A and B, respectively, while no voids were found in sample C. Although the ICP etching process is likely to alter the geometry of the voids, through an increase in their diameter, AFM images, shown in Figure 1d,e, are consistent with cross-sectional SEM results, as the voids in sample A appear larger than in sample B. Neglecting the deepest and widest features visible in Figure 1d, which likely correspond to the tallest initial voids that were thus exposed to a longer etching process, we measured void diameters lying in the 39–53 nm and the 39–51 nm range for samples A and B, respectively. Considering that these values are most certainly overestimated due to the etching process, and that those extracted from SEM images are probably underestimated, we conclude that the actual void diameter *d* likely lies in between these two estimates. A summary of the extracted void density and size for both samples is provided in the bottom part of Table 1.

With the purpose of probing the commonly found impurities in MOVPE-grown AlN epilayers, secondary-ion mass spectrometry (SIMS) was performed on the three layers at EAG Laboratories. In Figure 2, the impurity concentration profiles of oxygen (O), carbon (C) and hydrogen (H) are shown as a function of vertical position (*y*) in the corresponding epilayer, where *y* = 0 coincides with the AlN/sapphire interface. As can be noticed in Figure 2c, the concentration of hydrogen for *y* values comprised between 40 and 400 nm is higher in sample A than in the void-free sample C. Since this thickness range is in agreement with the position of the voids deduced from SEM images, the higher hydrogen content is likely related to the presence of saturated dangling bonds located at the AlN/void interfaces. For the same thickness range, oxygen and carbon atoms are also more abundant in sample A than in sample C, although sample C seems to have a slightly higher oxygen background in the top part of the layer. Somewhat similar features can be observed when comparing samples B and C, though the effect is far less pronounced due to the reduced size and the lower density of voids found in sample B. These signatures suggest that a higher concentration of impurities is found in the regions where voids are present, which is in agreement with other results from the literature.^34^

**Figure 2:**
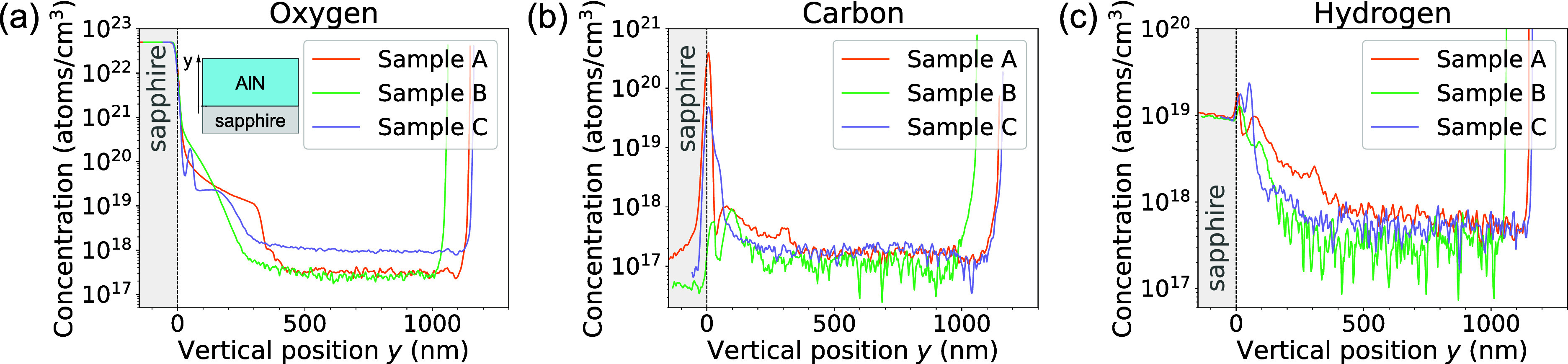
(a) to (c) SIMS concentration profiles of the main impurity species for the three AlN-on-sapphire epilayers under investigation. The higher hydrogen concentration level measured in sample A is consistent with the presence of voids in the first 400 nm of the layer.

### 2.2 Measurement of Propagation Losses

In order to quantify propagation losses occurring in samples A to C, WGs and MRRs were fabricated using an electron-beam lithography (EBL) step followed by full-depth AlN ICP dry etching and plasma-enhanced chemical vapor deposition (PECVD) of an SiO_2_ top cladding layer. Additional details on the fabrication process are reported in the Method section of the main text. A top-view false-color SEM image of a MRR with 60 μm radius prior to cladding deposition is presented in Figure 3a, while Figure 3b features a zoomed-in tilted-view image showing smooth AlN sidewalls. Finally, a cross-section SEM view of a WG facet is shown in Figure 3c, from which we measure an AlN sidewall angle of 80°, which is consistent with the values reported in the literature.^3,20^

**Figure 3:**
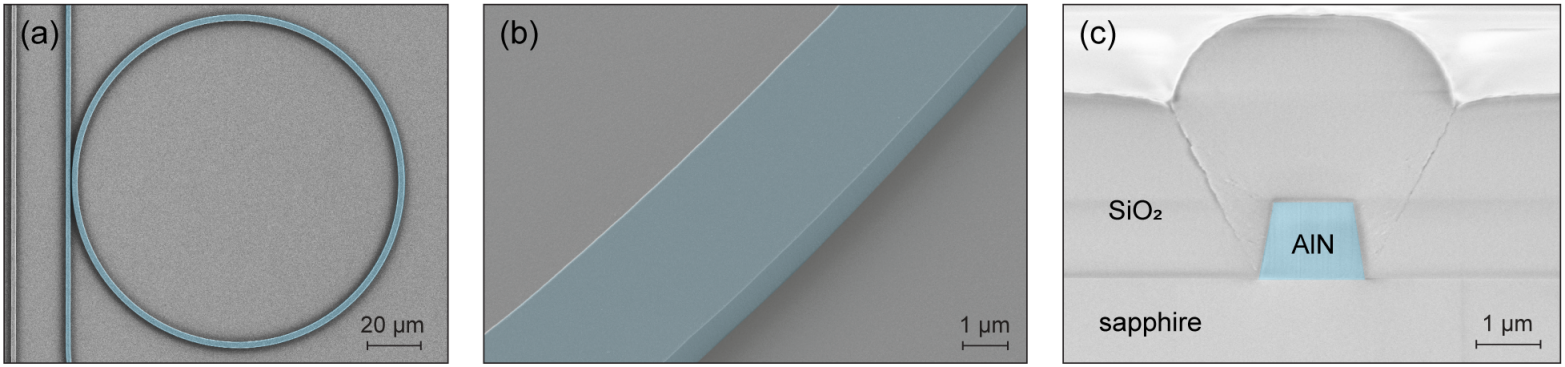
(a) False-color top view SEM image of an AlN MRR of 60 μm radius prior to SiO_2_ cladding deposition. (b) Enlarged bird’s eye view of an MRR showing a smooth sidewall. (c) False-color cross-section SEM image of a bus WG facet in sample C, revealing a sidewall angle of about 80°.

For WGs, propagation losses at 1550 nm were obtained using the cut-back method, i.e., by measuring the light power transmission for different waveguide lengths and fitting the data in dB scale with a linear model. For MRRs, light from a tunable laser emitting in the C-band was first coupled to bus WGs of upper width = 1.2 μm through a microlensed fiber, leading to evanescent coupling to rings of different widths and varying bus-to-ring gaps. The signal outcoupled from the bus WGs was collected either by an aspheric lens or a microlensed fiber. Transmission data were fitted with a Lorentzian function and the loaded quality factor (*Q*_L_) was deduced by dividing the wavelength at the resonance (λ_0_) by the fwhm of the resonance peak. The rings operated in the undercoupled regime, allowing the calculation of the intrinsic *Q* factor *Q*_int_ from the relationship:^3^

1 $$Q_{\mathrm{int}}=\frac{2Q_{L}}{1+\sqrt{T_{0}}}$$

where *T*_0_ is the normalized on-resonance transmission. Finally, we extracted the propagation loss coefficient α from^35^

2 $$\alpha =\frac{10\log_{10}\left(e\right)\cdot 2\pi n_{g}}{\lambda_{0}Q_{\mathrm{int}}}$$

where *n*_g_ is the group index of the resonant mode under consideration, obtained from finite-difference-eigenmode simulations performed with *Ansys Lumerical*, using refractive index data experimentally measured by spectroscopic ellipsometry on unprocessed AlN-on-sapphire epilayers.

Optical performance results obtained on waveguiding structures fabricated from the three types of epilayers are shown in Figure 4. Propagation losses in 1.4 μm-wide WGs from sample A are as high as 33.4 dB cm^–1^, as inferred from the slope of the linear interpolation shown in Figure 4a. Similar values are obtained for 2.3 μm-wide MRRs fabricated from the same type of epilayer, which yielded a *Q*_int_ value of 1.3 × 10^4^ for the TM_00_ mode, corresponding to α = 30.2 dB cm^–1^ (Figure 4b). Propagation losses in TE polarization resulted to be even higher, by ∼10 dB cm^–1^ above what was measured for TM polarization. On the contrary, sample B (which contains fewer and smaller voids than sample A) and sample C (which is void-free) both yield much lower values of propagation losses, as shown in the analysis of fundamental TE and TM modes for 1.8 μm-wide MRRs (Figure 4c–f). More specifically, *Q*_int_ can be as high as 2 × 10^6^ for the TE_00_ mode, (corresponding to losses <0.2 dB cm^–1^), and 1 × 10^6^ for the TM_00_ mode (corresponding to losses <0.4 dB cm^–1^).

**Figure 4:**
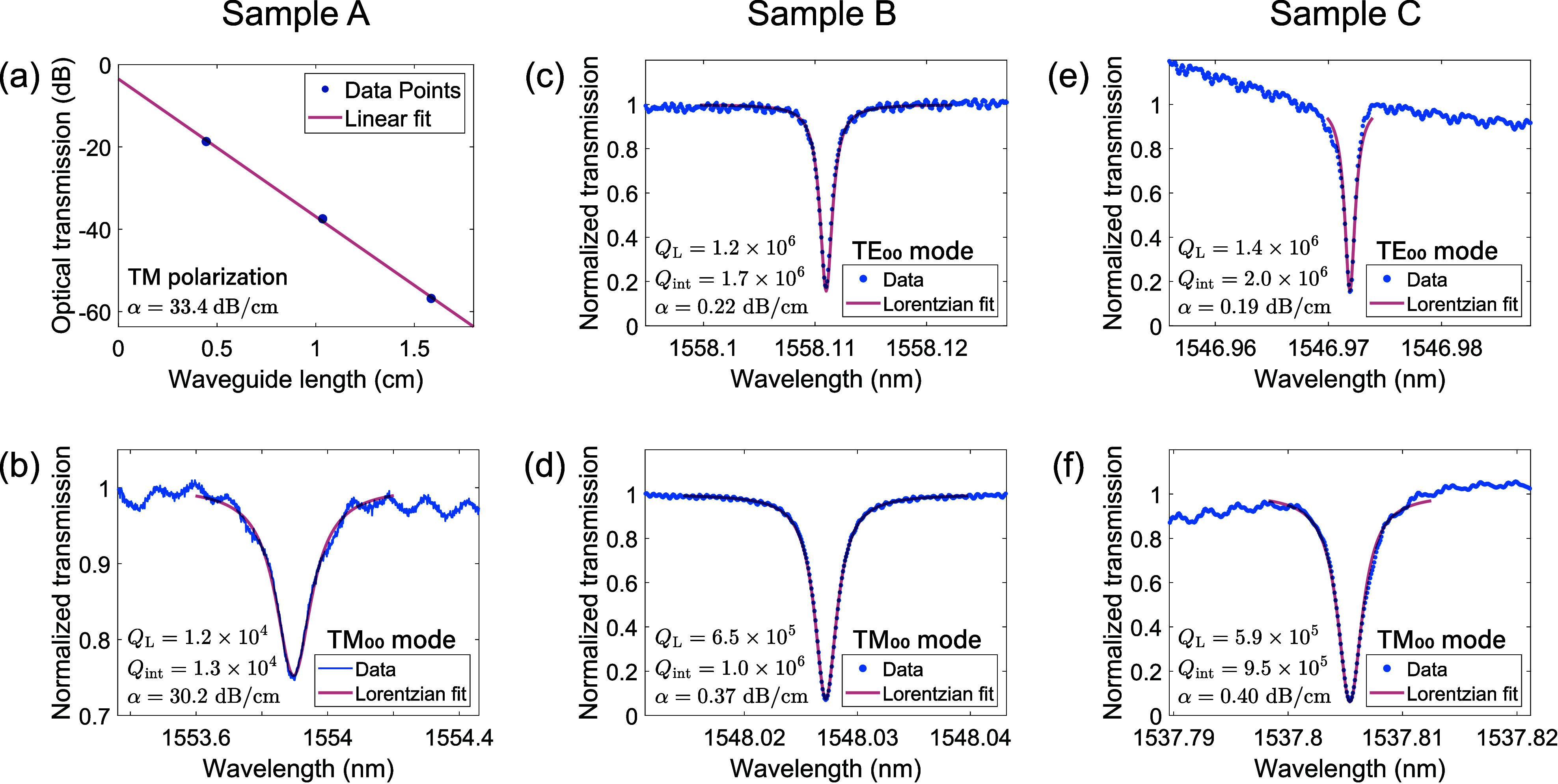
(a) Optical transmission in 1.4 μm-wide WGs fabricated from sample A, characterized at 1550 nm in TM polarization. (b) Transmission spectrum of a TM_00_ resonance in a 2.3 μm-wide MRR from sample A, showing optical losses around 30 dB cm^–1^. (c) to (f) Characterization of the fundamental TE and TM resonances in the C-band for 1.8 μm-wide MRRs fabricated from samples B and C. Rings have a radius of 60 μm and measurements were done in the undercoupled regime.

To better compare the three samples, several undercoupled ring resonances were analyzed, and the average values of *Q*_int_ and α, determined using eqs 1 and 2, respectively, are reported in Table 2, where errors represent the standard deviation of the resonance data set. As can be noticed, a decrease in the density and the size of the voids such as those present in samples A and B is sufficient to improve *Q*_int_ values by more than 2 orders of magnitude at telecom wavelengths. However, propagation losses reach the same magnitude for samples B and C, despite the complete absence of voids in the latter. We hypothesize that this limitation in both samples is rather due to optical loss channels introduced by our fabrication procedure. Our assumption is further confirmed when considering the data shown in Figure 5, for which *Q*_int_ values obtained for different ring widths in sample B are displayed. Indeed, *Q*_int_ is smaller for narrower ring WG widths because of the larger weight of sidewall scattering, while for rings wider than 1.8 μm, *Q*_int_ does not significantly increase with the ring width anymore. In particular, for the TE_00_ mode we did not obtain *Q*_int_ values higher than 1.8 × 10^6^ in sample B. Such a value remains smaller than the *Q*_int_ value of 2.8 × 10^6^, reported by Y. Sun et al. on MRRs fabricated on AlN epilayers from the same supplier, suggesting that our current results are limited by fabrication-related features.^36^

**Table 2:** Average *Q*_int_ Values and Propagation Losses Measured at 1550 nm in MRRs Fabricated from the Three Epilayers under Consideration. Data are Averaged over a Minimum of 5 Resonances per Dataset

		Sample A	Sample B	Sample C
TE_00_	*Q*_int_	Not available	(1.3 ± 0.3) × 10^6^	(1.4 ± 0.2) × 10^6^
	α (dB cm^–1^)	∼41	(0.29 ± 0.06)	(0.27 ± 0.04)
TM_00_	*Q*_int_	(1.3 ± 0.2) × 10^4^	(8.5 ± 1.2) × 10^5^	(8.7 ± 1.0) × 10^5^
	α (dB cm^–1^)	(31 ± 4)	(0.46 ± 0.08)	(0.44 ± 0.06)

**Figure 5:**
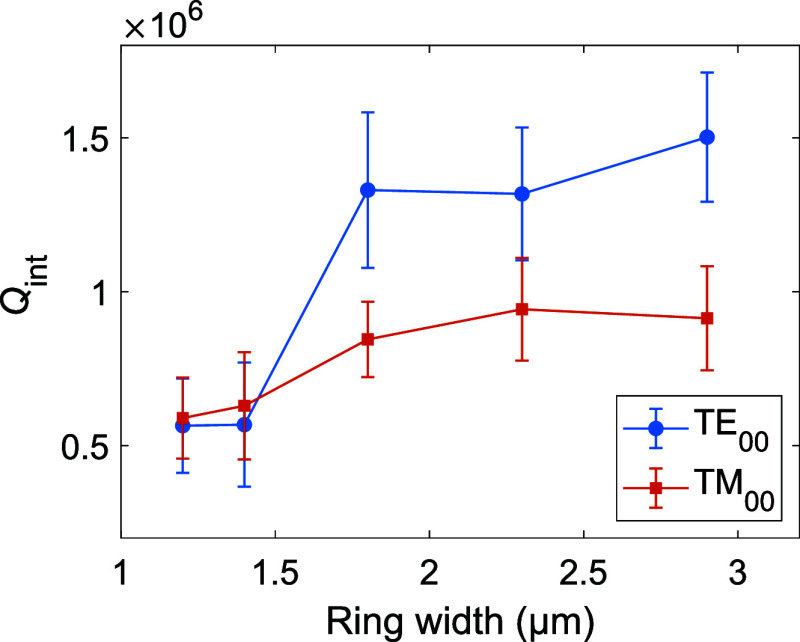
Experimentally measured mean *Q*_int_ value of the fundamental TE and TM resonances at λ ≃ 1550 nm in MRRs fabricated from sample B. The error bars correspond to the standard deviation for each MRR set. Microrings have a radius of 60 μm, an upper width that is varied from 1.2 to 2.9 μm, and a bus WG to ring resonator coupling gap chosen to ensure that MRRs operate in the undercoupled regime.

The explanation accounting for our lower quality factors compared to those obtained by Y. Sun et al.^36^ is probably multifactorial. First, we relied on silane-based PECVD for the SiO_2_ cladding, which is known to leave air pockets when narrow trenches, such as those present in the case of WG-resonator gaps, are filled, as documented in SI Section S2. This aspect plays an important role in MRR losses, as the vertically low lying air pocket can perturb the WG bus-microring coupling and be a non-negligible source of light scattering. In this regard, tetraethyl-orthosilicate-based PECVD, which was the cladding technique used in ref 36 is expected to provide better optical results due to its superior trench refilling capabilities.^37^ Furthermore, high-temperature (1200°C) annealing could be implemented in future works to further decrease optical losses occurring in the SiO_2_ cladding layer, due to a reduction in O–H bonds^38^ that are known to be a source of light attenuation at telecom wavelengths. Lastly, the dry etching parameters could be further optimized to achieve smoother sidewalls, although we do not expect sidewall roughness to be the main source of losses in our wider rings. Indeed, based on the analytical approach proposed in ref 3 roughness-induced scattering losses are expected to scale proportionally with the normalized average field intensity at the sidewalls (*I*_sw_). From the FDE simulations shown in SI Section S3, *I*_sw_ is found to decrease by a factor of 2 in a MRR when its width increases from 1.8 to 2.9 μm, leading to a corresponding 2-fold reduction in scattering losses. Since the quality factors shown in Figure 5 do not follow this trend, we can infer that propagation losses in our samples are not limited by sidewall roughness alone, for both TE and TM polarization.

In any case, regardless of these fabrication aspects, we can still conclude that when epilayers contain voids of non-negligible height (over 200 nm) and density (over 100 μm^–2^), they can lead to significant propagation losses at 1550 nm exceeding 30 dB cm^–1^, as observed in sample A.

### 2.3 Simulation of Void-Induced Scattering Losses

In order to confirm that voids are responsible for volumetric scattering and can be a source of significant propagation losses in fully MOVPE-grown AlN photonic structures, we performed FDTD simulations using *Ansys Lumerical* (*Ansys Lumerical* FDTD v. 8.30.3536 from Lumerical Inc.). Specifically, we investigated propagation losses occurring in the telecom C-band for the fundamental TE and TM modes of an AlN-on-sapphire WG (height: 1.1 μm, upper width: 1.4 μm) for a total length *z* of 90 μm, in the absence and in the presence of voids. For the sake of simplicity, voids were modeled as square parallelepipeds, randomly generated inside the AlN WG, with sizes in agreement with the experimentally measured values for sample A, as reported in Table 1. Hence, the height *H* is randomly distributed in the 40–300 nm range, while the bottom coordinate *y*_min_ lies in the 40–200 nm range, the areal density is fixed to *N* = 120 μm^–2^, and we consider different diameter *d* values. Perfectly matched-layer boundary conditions were adopted in order to absorb the scattered power reaching the edges of the simulation window. A fine mesh of 10 nm was used in the bottom part of the WG, where voids are present, while coarser meshes were used in the rest of the WG region and in the remaining parts of the simulation domain. Given the subwavelength dimensions of the voids compared to the optical wavelength, this simplified void representation is expected to capture the main scattering effects, while keeping the computational cost manageable. Additional details on the simulation settings and the convergence can be found in the SI Section S4.

In Figure 6a,b, we show a two-dimensional (2D) top-view map of the electric field modulus |*E⃗*| at WG midheight in the absence and in the presence of voids, respectively. While the optical mode appears stable in the absence of defects, a non-negligible amount of power escapes from the WG due to scattering when voids are present.

**Figure 6:**
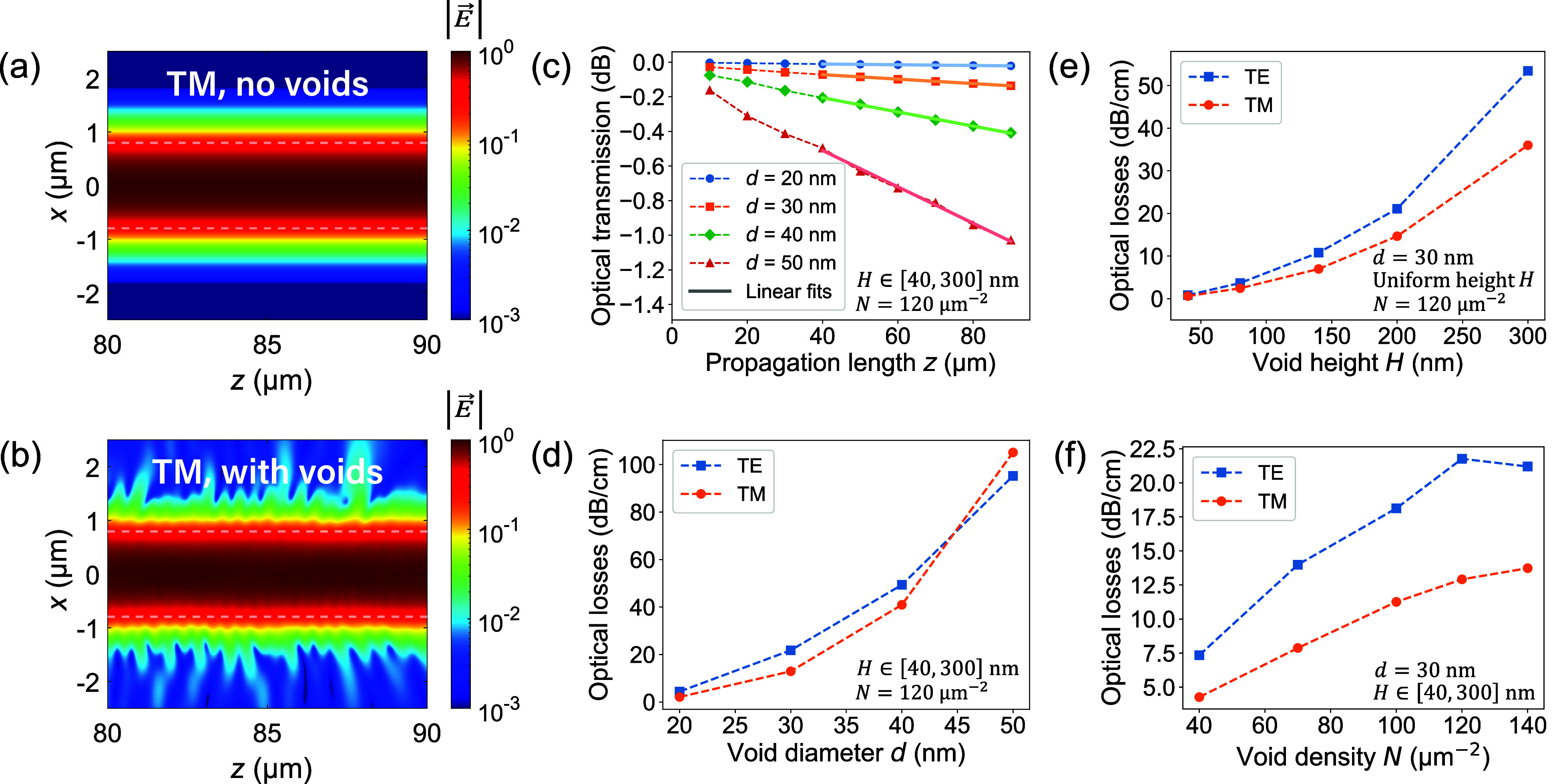
FDTD simulations of light transmission at 1550 nm for AlN straight WGs with different void geometries (height: 1.1 μm, upper width: 1.4 μm). (a) and (b) 2D top-view normalized field |*E⃗*| map at WG midheight in the absence and in the presence of voids, respectively, showing the signature of scattering induced by the latter. The WG geometry is delimited within the two dashed lines. (c) Power transmission as a function of propagation length for different void diameters *d* at a fixed void density *N* = 120 μm^–2^, in TM polarization. Data in decibels are fitted with a straight line to extract propagation losses. (d) Simulated optical losses as a function of *d* for both polarizations. (e) Simulated optical losses as a function of void height *H* for *d* = 30 nm. (f) Simulated optical losses as a function of void density *N* for *d* = 30 nm.

We monitored the optical transmission every 10 μm along the propagation direction *z* for different values of the void diameter *d*. Optical losses were extracted from the linear fit of the slope in dB scale, where the first three data points were excluded to minimize their misestimate because of a potentially higher collection of the scattered field in the first power monitors. As an illustrative example, we report in Figure 6c the results obtained for TM polarization for which a strong dependence of transmission on *d* is observed. The optical loss values obtained using this method for both TE and TM input polarizations are shown in Figure 6d. With the exception of the data points computed at *d* = 50 nm, TE loss values are larger than TM ones, in agreement with the experimental results obtained on sample A (cf. Table 2). We see that for a *d* value of 30 nm, a reasonable assumption given the two estimates of the void diameter deduced from cross-section SEM and top-view AFM images, we derive losses of (21.8 ± 0.4) dB cm^–1^ and (12.9 ± 0.1) dB cm^–1^ for TE and TM polarizations, respectively. Although they are on the same order of magnitude, the simulated values are smaller than experimentally measured losses of ≃41 dB cm^–1^ (TE) and (31 ± 4) dB cm^–1^ (TM) in sample A. However, given the strong dependence of the computed losses on the void size, such a difference could stem from an experimentally underestimated value of the size of the voids and their density. We also recall that higher concentrations of O, C and H impurities are found in the void region, as shown in Figure 2. Therefore, part of the losses we observed in sample A could also be due to vibrational modes of impurity-related bonds, similarly to the losses induced by N–H and Si–H bonds in silicon nitride WGs.^39,40^

Keeping *d* = 30 nm and *N* = 120 μm^–2^ as reference values, we then assigned to all voids a uniform height value to study its impact on propagation losses. Thus, as can be seen in Figure 6e, height seems to play a role as important as the diameter in the magnitude of those losses. Finally, simulations carried out for different void densities *N* are shown in Figure 6f, which also indicate an increase in the propagation losses with *N*, as could be anticipated.

Given the remarkable difference in void density and most importantly in size between samples A and B, it is not surprising that we could not observe the impact of voids in sample B from our measurements led at 1550 nm. However, such small voids are likely to play a non-negligible role when aiming for short-wavelength applications, e.g., in the visible and in the UV range, because the weight of volumetric scattering losses is expected to scale as λ^–4^. Moreover, in order for a WG to remain in the single-mode regime at shorter wavelength, thinner layers are required. In such a case, as shown in Figure 7a,c, most of the optical mode power will reside in the region occupied by voids, leading to enhanced scattering losses compared to the situation experienced by single-mode WGs in the telecom C-band.

**Figure 7:**
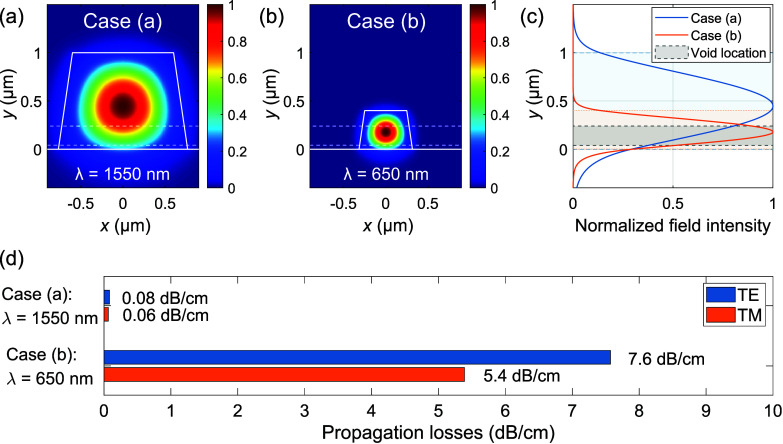
Simulated TE_00_ normalized field intensity (|*E⃗*|^2^) maps computed for two different cross-section WG geometries: (a) at λ = 1550 nm in a 1.2 × 1.0 μm^2^ WG and (b) at λ = 650 nm in a 0.5 × 0.4 μm^2^ WG, where in each case the WG geometry has been chosen such that the devices operate in the single-mode regime for TE polarization. (c) Field intensity profile at the WG center for field intensity maps (a) and (b), showing a larger overlap of the field intensity distribution with the void region for case (b). (d) Losses extracted from FDTD simulations for both cases, in the presence of “small″ voids in the layer.

To verify these hypotheses, we carried out an additional set of FDTD simulations considering smaller voids, similar to those observed in sample B. Voids were randomly generated in the waveguide with the following parameters: *d* = 20 nm, *H* = 40 nm, *N* = 40 μm^–2^, and *y*_min_ in the 40–200 nm range. Two cases were analyzed: a 1.2 × 1.0 μm^2^ WG pumped at 1550 nm (case (a)), and a 0.5 × 0.4 μm^2^ WG pumped at 650 nm (case (b)). Results are reported in Figure 7d, where we can observe how small voids are only responsible for negligible propagation losses at 1550 nm, lower than 0.08 dB cm^–1^. This explains why we could not detect significant differences in propagation losses between sample B and the void-free sample C, as losses in our structures are likely dominated by fabrication-related aspects. However, the same void distribution is expected to cause significant propagation losses at 650 nm, namely 7.6 dB cm^–1^ in TE polarization, and 5.4 dB cm^–1^ in TM, due to a combination of increased scattering at shorter wavelengths and a higher portion of the modal field interacting with voids in this second case (as shown in Figure 7c). The exact values obtained from these sets of simulations on smaller voids should be regarded as a semiquantitative indication, since the results depend on a wide range of void parameters that are difficult to determine accurately. Nevertheless, they are useful for illustrating how even voids with a negligible impact at telecom wavelengths can become a major source of propagation losses within the visible range.

For all these reasons, the presence of voids, even of small size, is clearly detrimental for PIC applications as they can act as a major source of losses in AlN photonic structures intended for short-wavelength operation. Moreover, the losses of 5–8 dB cm^–1^ we predicted from simulations at 650 nm are consistent with some of the values reported in literature for AlN microring resonators operating in the visible,^41^ indicating that voids could likely be one of the limiting factors in current state-of-the art AlN photonics in the visible.

Therefore, optimizing the growth process to reduce or even suppress their presence can be an effective strategy for improving the optical performance of AlN-on-sapphire PICs in this wavelength range. In this regard, being entirely void-free, the present hybrid AlN epilayers grown on a sputtered AlN buffer emerge as a promising solution for visible and UV applications with the potential to overcome current state-of-the-art values for this platform.^3^

### 2.4 Nonlinear Measurements on Hybrid AlN-on-Sapphire Layers

To illustrate the potential of the hybrid sample C for nonlinear optical applications, we investigated the on-chip SHG and SCG in WGs. From the nonlinear response of the dispersion-engineered WGs, we can infer information about the nonlinear optical parameters of the material, the quality of the epilayers, and fabrication process. The attenuation of the nonlinearly generated signal in different frequency bands can show the influence of wavelength-dependent losses related to the presence of voids, sidewalls, and impurity-induced scattering, as well as cladding absorption. To this end, we tested the WGs fabricated from samples B and C following the fabrication process used for MRRs. The WG length was set at 4.5 mm, the height was fixed by the epilayer thickness, and the width was varied to tailor the modal dispersion.

SHG can occur only with a TM-polarized pump that aligns with the strongest component of the AlN nonlinear susceptibility tensor.^19,42^ Figure 8a reports efficient SHG in 1.25 μm-wide WGs from the hybrid sample C while sweeping the TM pump in the telecom range. The peak corresponds to the intermodal phase-matching (PM) between the TM_00_ pump and the TM_20_ mode at the second harmonic (SH), and its position is strongly dependent on the exact WG geometry, as we show in the mode simulations reported in SI Section S5. Moreover, the SH peak position can be finely tuned by varying the sample temperature. Indeed, as shown in Figure 8a, by changing the sample temperature from 20°C (*T*_1_) to 83°C (*T*_2_), the SH peak shifts to longer wavelengths.

**Figure 8:**
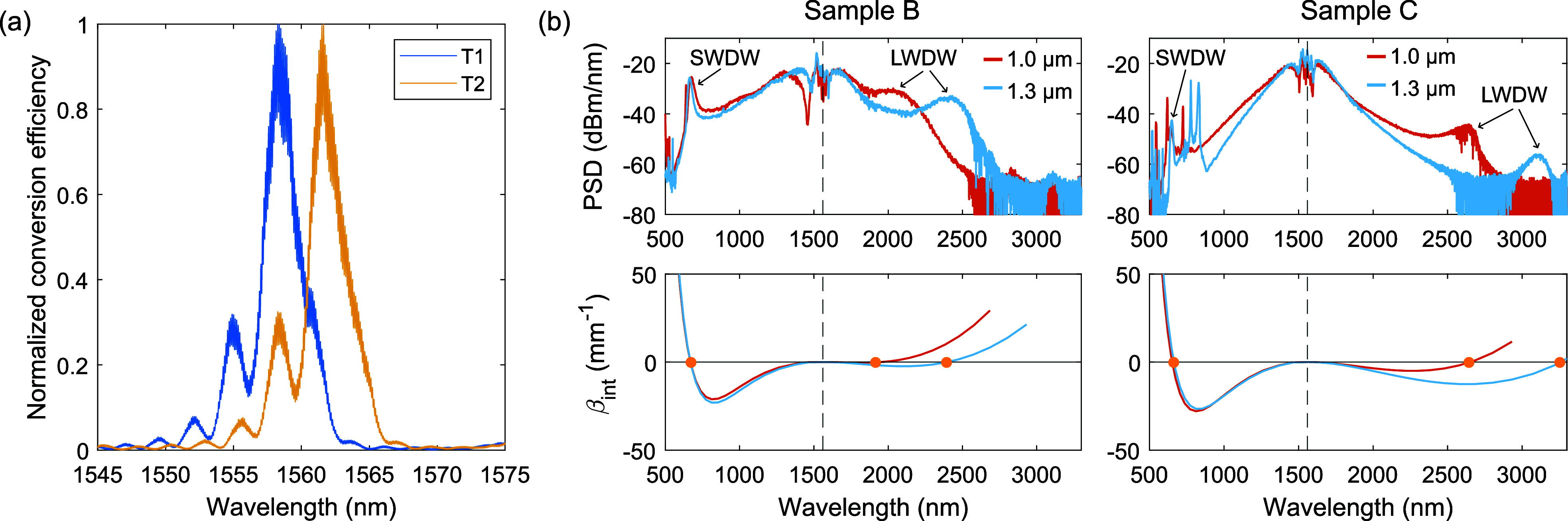
(a) Normalized conversion efficiency of the SH power generated from a WG on sample C while sweeping the pump wavelength in the telecom range. The experiment is run at two different temperatures (*T*_1_ = 20°C, *T*_2_ = 83°C). (b) Comparison between the SC spectra generated by 1 μm- and 1.3 μm-wide WGs fabricated on sample B (top left) and C (top right), respectively. The TM-polarized pump wavelength is set at 1.56 μm (dashed line) and the positions of the SWDW and LWDW are indicated. In the bottom panels the corresponding integrated dispersions (β_int_) are plotted. The phase-matching condition for the SWDW and the LWDW is indicated by yellow dots.

We then tested SCG through soliton dynamics and dispersive wave (DW) generation obtained by pumping the WGs with a femtosecond laser in the anomalous dispersion region, i.e., where β_2_ < 0, with β_2_ the second derivative of the wavenumber β with respect to the angular frequency ω. The pump has a central wavelength of 1560 nm, TM polarization, a pulse duration of 46 fs, and a repetition rate of 100 MHz. Light was coupled into the chip and collected at the output through an aspheric and an achromatic lens, respectively, and sent to optical spectrum analyzers covering the spectral range from the visible to the mid-IR. The resulting spectra for 1.0 μm- and 1.3 μm-wide WGs are shown in the top panels of Figure 8b, for samples B (left) and C (right). These are consistent with results obtained previously with similar geometries and power conditions.^12^ The supercontinuum (SC) coverage is primarily limited by the spectral positions of the long-wavelength DW (LWDW) and the short-wavelength DW (SWDW), located at phase-matched wavelengths between the solitons and the linear waves in the normal dispersion regime (β_2_ > 0). This PM condition depends on the WG cross-section, leading to a shift of the DW when WGs with the same height but different widths (same epilayer) or with the same width but different heights (different epilayers) are compared. The bottom panels of Figure 8b show the corresponding integrated dispersion, given by β_int_ = β­(ω_DW_) – β­(ω_s_) – (ω_DW_ – ω_s_)/*v*_g,s_, with ω_DW_ and ω_s_ the angular frequency of the DW and soliton, and *v*_g,s_ the soliton group velocity. Applying the β_int_ = 0 condition, we can find the DW position, indicated on the plots by yellow dots. The good correspondence between the experimental and simulated positions of the DWs validates the chromatic dispersion profile that was considered and provides valuable insight into the precision of the WG dimension control. The SC extends further in the short wavelength side in sample C, compared to sample B, showing promising properties for visible light applications. The dispersion of the WGs of sample C, which are slightly thicker than those made from sample B, is also more favorable for pushing the LWDW deeper into the mid-IR. However, our simulations indicate that the larger thickness of sample C relative to sample B would lead to a lower DW generation efficiency, as a result of significantly larger |β_int_| values and a reduced spectral overlap between the soliton and its corresponding DW. Experimentally we also see a reduction in the LWDW power, dominated by the above-mentioned effects. Optimization of the generation efficiency in thicker layers would require additional tuning of the pump wavelength, as shown in silicon nitride^43,44^ and AlN^45^ WGs.

Overall, these results demonstrate an efficient nonlinear response, control over the WG dispersion and dimensions, and confirm the suitability of the present hybrid AlN-on-sapphire epilayers as an integrated platform for harmonic and broadband light generation. The low transmission losses achieved through optimized growth and nanofabrication process, together with the ability of engineering the WG dispersion by adjusting their dimension, suggest the possibility of pushing the spectral coverage deeper into both the UV and mid-IR.

## 3 Conclusion

In conclusion, we experimentally showed the detrimental impact of voids on the optical performance of AlN-on-sapphire WGs and MRRs operating at 1550 nm. Complementary light propagation FDTD simulations done for AlN straight WGs with and without voids unambiguously show their key role on scattering losses, which are mostly dependent on their size and density. Moreover, our simulation results indicate that even small voids, which are expected to cause negligible propagation losses at telecom wavelengths, have a noticeable impact on light propagation at shorter wavelengths, suggesting that voids may represent one of the potential limiting factors in current state-of-the art AlN photonics in the visible range.

Finally, we showed that a hybrid approach consisting of an MOVPE-grown AlN layer deposited on a thin sputtered AlN buffer leads to void-free epilayers characterized by low propagation losses in the telecom C-band, as confirmed by *Q*_int_ values up to 2 million measured at 1550 nm in MRRs. Such hybrid AlN epilayers also proved suitable for high-power nonlinear optical applications, as verified by SHG and broadband SCG measurements. Furthermore, since void-related scattering is expected to be more impactful at short wavelengths, the void-free nature of these hybrid AlN epilayers makes them appealing candidates for low-loss PICs operating in the visible and UV ranges, with the potential to advance the state-of-the-art performance of this platform.

## 4 Method

### 4.1 Growth of AlN Epilayers

Sample A was grown in an Aixtron 200/4 RF-S horizontal reactor on a *c*-plane single-side polished sapphire substrate with a miscut angle of 0.2° toward the *m*-axis. A 3 nm-thick AlN nucleation layer was first grown at a temperature of 700°C to initiate the Al polarity. AlN growth was then performed at a temperature of 1100°C, up to a final thickness of approximately 1.1 μm. Sample B is a 1.0 μm-thick commercially available epilayer on *c*-plane single-side polished sapphire from DOWA Electronics Materials Co., Ltd. For sample C, a 50 nm-thick AlN buffer layer was first deposited via sputtering by Evatec AG onto a sapphire substrate identical to that of sample A. MOVPE regrowth was then performed using the same reactor and a growth procedure at 1100°C similar to that of sample A, up to a total thickness of about 1.1 μm.

### 4.2 Device Fabrication

Waveguiding structures were patterned onto the AlN epilayers using a 100 kV EBL machine (Raith EBPG5000+) and the negative-tone FOx-16 hydrogen silsesquioxane (HSQ) resist. A thin titanium (Ti) layer was evaporated onto the samples prior to resist coating to facilitate charge dissipation. The pattern was transferred from the FOx-16 resist to the AlN layers by means of a Cl_2_/BCl_3_/Ar-based ICP dry-etching process, with an AlN:FOx-16 etching selectivity of 2.2:1. After etching, the Ti and HSQ residues were removed with a buffered oxide etchant solution and the AlN structures were then cladded with 3 μm of PECVD-deposited SiO_2_. Lastly, the chips were cleaved along the AlN *m*-plane for edge coupling using the dice-and-cleave technique.^46^ For simple WGs in sample A (Figure 4a), facet polishing was also employed to further even out coupling losses across the chip.

### 4.3 Device Characterization

The fabricated PIC devices were tested with a Toptica CTL 1550 tunable laser. Light was coupled to the chip with a microlensed fiber (spot size = 2.3 μm) and collected either with a second microlensed fiber (when measuring the WG losses with the cut-back method) or with a free-space objective (for MRR characterization) and sent to an infrared photodetector. For samples B and C, given the narrow MRR resonance line widths, the wavelength scan was performed through a voltage sweep on the laser piezoelectric actuators, calibrated with an electro-optical modulator and a signal generator in the GHz range.

## Supplementary Material



## Notes

Additional material characterization of the AlN epilayers used for this work, including the formation of air pockets subsequent to PECVD SiO_2_ cladding deposition; details related to both sidewall scattering losses occurring in MRRs and FDTD simulations of void-induced scattering losses; simulations of phase matching for SHG (PDF)
