# Supplementary material for: Sputtered AlN Buffer Layer for Low-Loss Crystalline AlN-on-Sapphire Integrated Photonics
Source: ACS Photonics. 2026 Feb 10;13(5):1388–98. doi: 10.1021/acsphotonics.5c02661 (PMC12964529; doi:10.1021/acsphotonics.5c02661)
Supplement: Supplementary file 1 [file ph5c02661_si_001.pdf]

Supporting Information for: Sputtered AlN Buffer Layer for Low-Loss Crystalline AlN-on-Sapphire Integrated Photonics

Samuele Brunetta[*,†], Samantha Sbarra[‡], Brandon Shuen Yi Loke[‡], Jean-François Carlin[†], Nicolas Grandjean[†], Camille-Sophie Brès[‡], and Raphaël Butté[†]

[†]Laboratory of Advanced Semiconductors for Photonics and Electronics, Ecole Polytechnique Fédérale de Lausanne (EPFL), CH-1015 Lausanne, Switzerland

[‡]Photonic Systems Laboratory, Ecole Polytechnique Fédérale de Lausanne (EPFL), CH-1015 Lausanne, Switzerland

[*]Corresponding author: `samuele.brunetta@epfl.ch`

Contents

S1 Additional material characterization of AlN-on-sapphire epilayers **S2**

S2 Bus-microring gap filling capability of silane-based PECVD **S5**

S3 Evaluation of sidewall scattering losses in microring resonators **S6**

S4 3D-FDTD simulation settings and convergence **S7**

S5 Simulations of phase matching for SHG **S9**

S1 Additional material characterization of AlN-on-sapphire epilayers

In this section we provide additional material characterization data for the three aluminum nitride (AlN) epilayers used in this work. The samples were all grown on 2-inch *c*-plane sapphire substrates by metalorganic vapor-phase epitaxy (MOVPE). Sample A was grown in house using a full-MOVPE process, which includes a thin AlN nucleation layer grown at lower temperature to facilitate the start of the heteroepitaxial growth process. Sample B is a 1.0 µm-thick commercially available epilayer purchased from DOWA Electronics Materials Co. Ltd. For sample C, a hybrid growth process was used, which consists of the MOVPE growth of an approximately 1.1 µm-thick AlN layer on a 50-nm-thick sputter-deposited AlN buffer layer on *c*-plane sapphire provided by Evatec AG. Additional details are provided in the Method section of the main text.

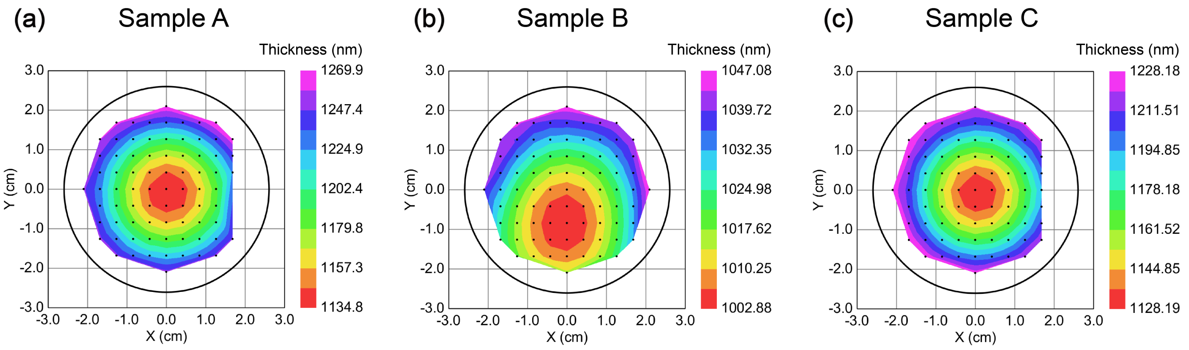

Figure S1: (a) to (c) 2D thickness maps of epilayers A, B and C, respectively, measured by spectroscopic ellipsometry.

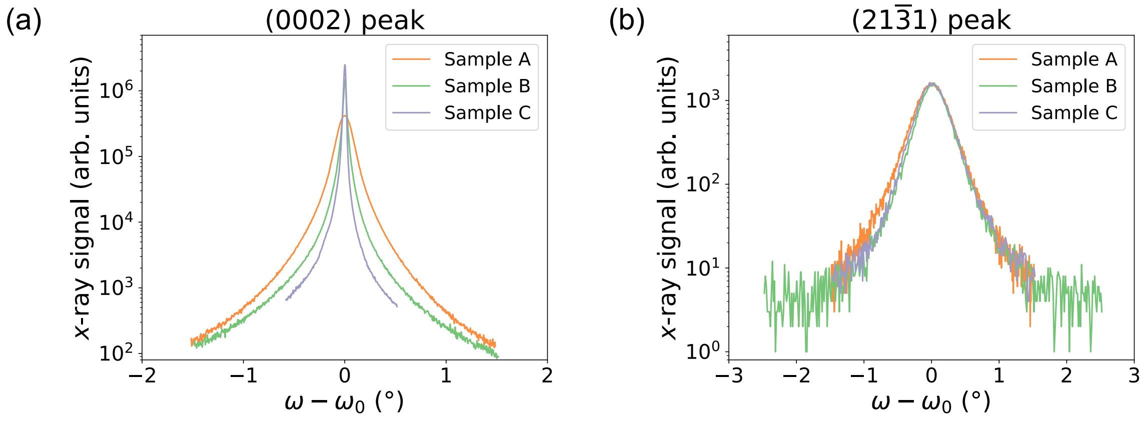

Figure S2: XRD rocking curves of the three AlN epilayers under consideration. (a) Results for the (0002) symmetric peak. (b) Results for the $(21\bar{3}1)$ asymmetric peak.

In Figures S1(a) to S1(c), we show the two-dimensional (2D) AlN thickness maps of epi-

layers A, B and C, respectively, obtained by spectroscopic ellipsometry using a Woollam RC2 tool. Single-point measurements at the wafer center, performed in the 300-2500 nm wavelength range and at incident angles of 55°, 65° and 75°, were used to determine the refractive index curve of the layer by fitting the data with a uniaxial Sellmeier model. Then, scans at a 55° angle were taken across the whole wafer in order to extract the thickness map using the refractive index data obtained with the single-point measurements. In all the samples, the AlN layer thickness increases from the wafer center toward the edges. However, while the thickness variation across the wafer is about 100 nm for the in-house grown samples A and C, the commercially available sample B exhibits thickness variations less than 50 nm. These features are most likely stemming from differences in the growth parameters and the reactor geometries (single 2-inch wafer capability for the in-house horizontal reactor). Thickness variation can be a critical parameter in applications that require precise dispersion engineering, such as second-harmonic generation and supercontinuum generation. In contrast, it plays a much less significant role when the objective consists in extracting propagation losses from waveguides (WGs) or microring resonators (MRRs). Moreover, for individual devices fabricated from the full wafer (e.g., our chips typically have dimensions of $4.5 \times 10\,\mathrm{mm}^2$), the local thickness variation is much smaller, and the design of photonic components—such as WGs—can be adjusted on each single chip based on the 2D thickness map of the epilayer.

X-ray diffraction (XRD) data for the (0002) symmetric and $(21\bar{3}1)$ asymmetric peaks are shown in Figures S2(a) and S2(b), respectively. While the three epilayers show little difference in the asymmetric peak, sample A has a broader symmetric peak than samples B and C, suggesting that sample A has a poorer crystalline quality and a higher degree of off-plane crystal orientation than both samples B and C (which are instead comparable to each other). The values of the full width at half maximum (FWHM) for both peaks in the three samples are those reported in Table 1 of the main text.

Surface roughness and topography were probed by means of atomic force microscopy (AFM). In Figures S3(a), S3(b) and S3(c), we report $2 \times 2\,\mu\mathrm{m}^2$ AFM surface scans of samples A, B and C, respectively, whose extracted value of the root mean square (RMS) surface roughness remains lower than 0.2 nm for all the samples. In the inset of Figure S3(c), we show a $1 \times 1\,\mu\mathrm{m}^2$ map of the 50 nm-thick sputtered AlN buffer layer prior to MOVPE regrowth. The layer has an RMS surface roughness of 0.72 nm, which is higher than the value of 0.09 nm measured for the full layer after regrowth. This means that the MOVPE growth process manages to recover the rougher surface of the sputtered layer and produces a remarkably smooth surface in the final layer.

Although all the samples present smooth surfaces at small scale, larger AFM scans such as shown in Figures S3(d) to S3(f) reveal the presence of some hexagonal hillocks and faceted macrosteps in samples A and C. These hillocks, which are not found in the commercially available sample B, are present with densities of $1.1 \times 10^7\,\mathrm{cm}^{-2}$ in sample A and $2.1 \times 10^7\,\mathrm{cm}^{-2}$ in sample C, and their height ranges between 5 and 30 nm. Taking hillocks into account, the

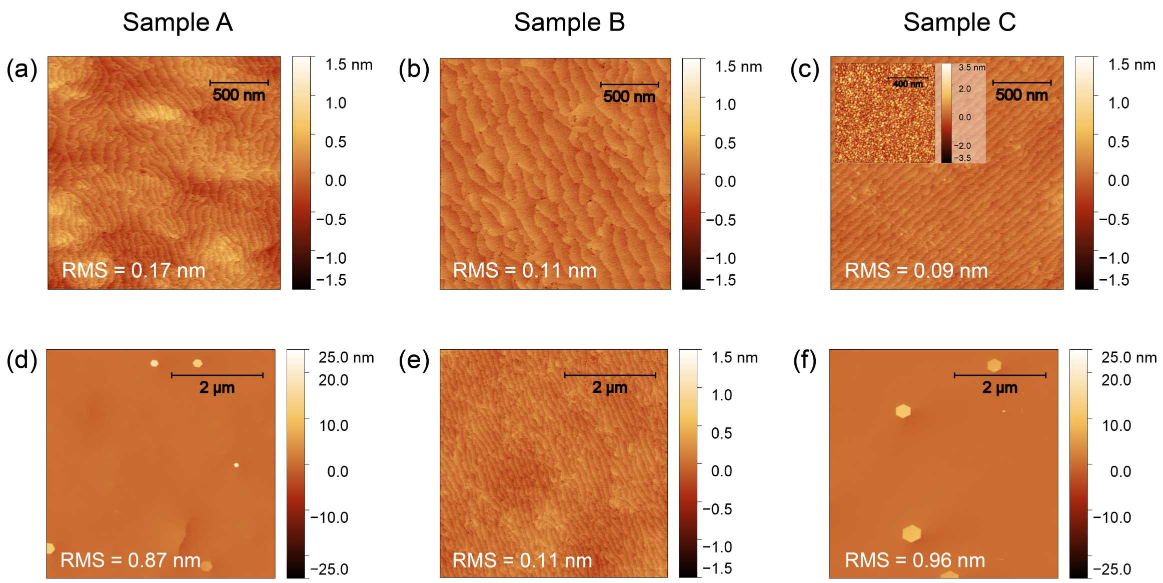

Figure S3: (a) to (c) $2 \times 2\,\mu m^2$ AFM surface scans of epilayers A, B and C, respectively, with the extracted value of the RMS surface roughness. The inset in Figure S3(c) shows a $1 \times 1\,\mu m^2$ surface scan of the 50 nm thick AlN sputtered buffer layer prior to MOVPE regrowth, yielding an RMS surface roughness of 0.72 nm. (d) to (f) $5 \times 5\,\mu m^2$ AFM surface scans of epilayers A, B and C, respectively, revealing the presence of hexagonal hillocks and faceted macrosteps in samples A and C.

RMS surface roughness of samples A and C is higher, as shown in Figures S3(d) and S3(f), but still below the 1 nm threshold. Hillocks in MOVPE grown AlN are commonly associated with several factors including growth temperature, the presence of γ-AlON islands, the V/III ratio and surface preparation.[1,2] However, optimizing the growth recipe can be a complex and time-consuming process and the presence of hillocks did not visibly impact propagation losses at 1550 nm in our devices, as we were able to fabricate MRRs from sample C with an intrinsic quality factor (Q_{int}) up to 2.0×10^6, corresponding to optical losses of 0.19 dB cm^{-1}. Therefore, we decided not to focus our attention on minimizing or even suppressing hillocks in this work. However, hillocks could potentially cause some non-negligible surface scattering losses at shorter wavelengths. Hence, their presence should likely be minimized in AlN epilayers intended for such applications.

Biaxial stress was estimated by means of micro-Raman spectroscopy using a Renishaw in-Via Reflex Raman Confocal microscope. In Figure S4(a), we show Raman spectra recorded on the three epilayers using continuous wave (cw) 532 nm laser excitation. The zoomed-in results for the AlN E_2(high) mode are shown in Figure S4(b). By fitting the data with a Lorentzian curve we extracted an E_2(high) peak center ω of 654.6 cm^{-1}, 657.8 cm^{-1} and 654.9 cm^{-1} for samples A, B and C, respectively. The biaxial stress σ_{xx} can be calculated using the relationship:[3]

$$\sigma_{xx} = \frac{\omega - \omega_0}{k}, \tag{S1}$$

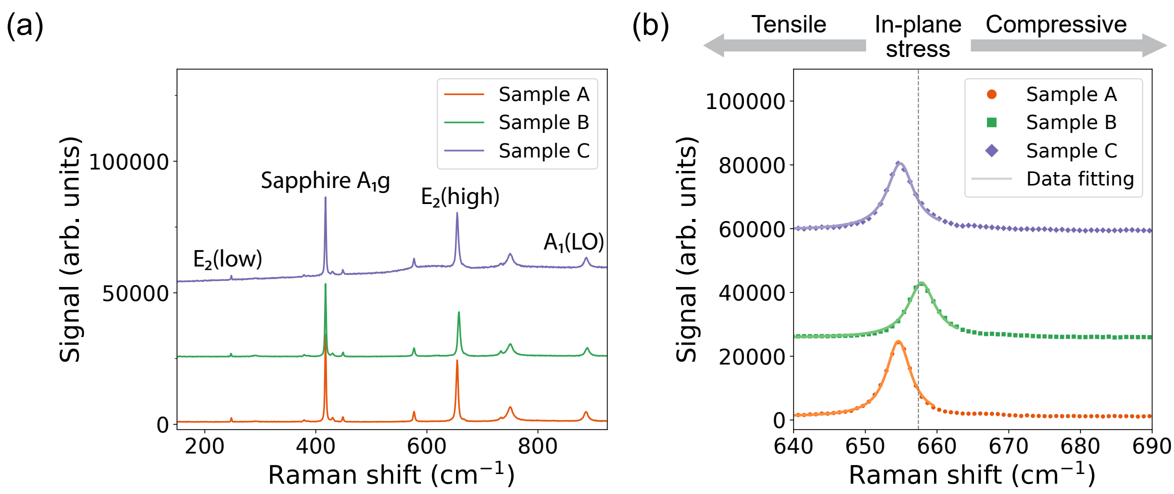

Figure S4: (a) Raman spectra of the three epilayers recorded using cw 532 nm laser excitation. (b) Raman data for the AlN E_2(high) mode, with their corresponding Lorentzian fit, showing different stress conditions in the three samples.

where $\omega_0 = 657.4\,\mathrm{cm}^{-1}$ is the E_2(high) peak position of strain-free aluminum nitride[4] and $k = -4.04\,\mathrm{cm}^{-1}\,\mathrm{GPa}^{-1}$ is the biaxial stress coefficient.[5] Using Eq. (S1) we obtain values of σ_{xx} of 0.69 GPa, −0.11 GPa and 0.62 GPa for samples A, B and C, respectively. These results indicate that, while the commercially available sample B is under slight compressive stress, samples A and C are subjected to tensile stress, which could likely explain the presence of a few isolated cracks in both samples A and C.

S2 Bus-microring gap filling capability of silane-based PECVD

As mentioned in the main text, the SiO_2 cladding layer was deposited by silane-based plasma-enhanced chemical vapor deposition (PECVD). This technique is known to leave air pockets when filling narrow trenches such as the bus-microring gap. Since most of the undercoupled MRRs we characterized in samples B and C have gaps ranging between 500 and 800 nm, we report in Figure S5 false-color cross-section scanning electron microscopy (SEM) images showing the PECVD trench filling capability in this range of gaps. As can be observed, an air pocket of non-negligible size is always present, even for the largest gap we considered. This air pocket can perturb the bus-microring coupling and act as a source of scattering. Therefore, it is probably one of the main factors currently limiting the performance of the fabricated devices. Potential improvements could be obtained by using tetraethyl-orthosilicate-based PECVD, which has superior trench refilling capability compared to silane-based PECVD.[6,7] However, this aspect goes beyond the scope of this work and is left for future investigations.

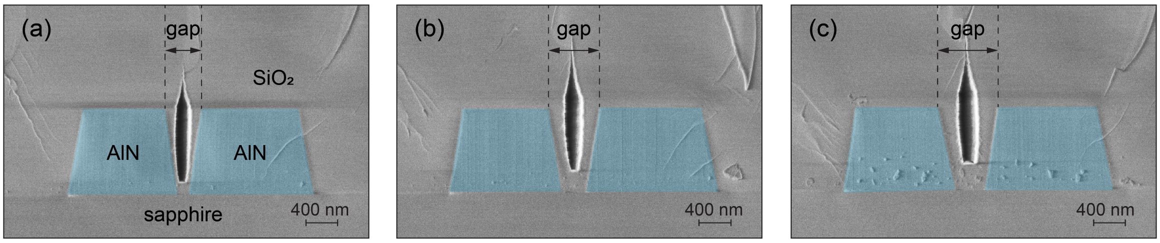

Figure S5: Cross-section SEM images showing the PECVD SiO_2 trench filling capability for different bus-microring gap configurations on sample B. (a) gap = 500 nm. (b) gap = 700 nm. (c) gap = 800 nm.

S3 Evaluation of sidewall scattering losses in microring resonators

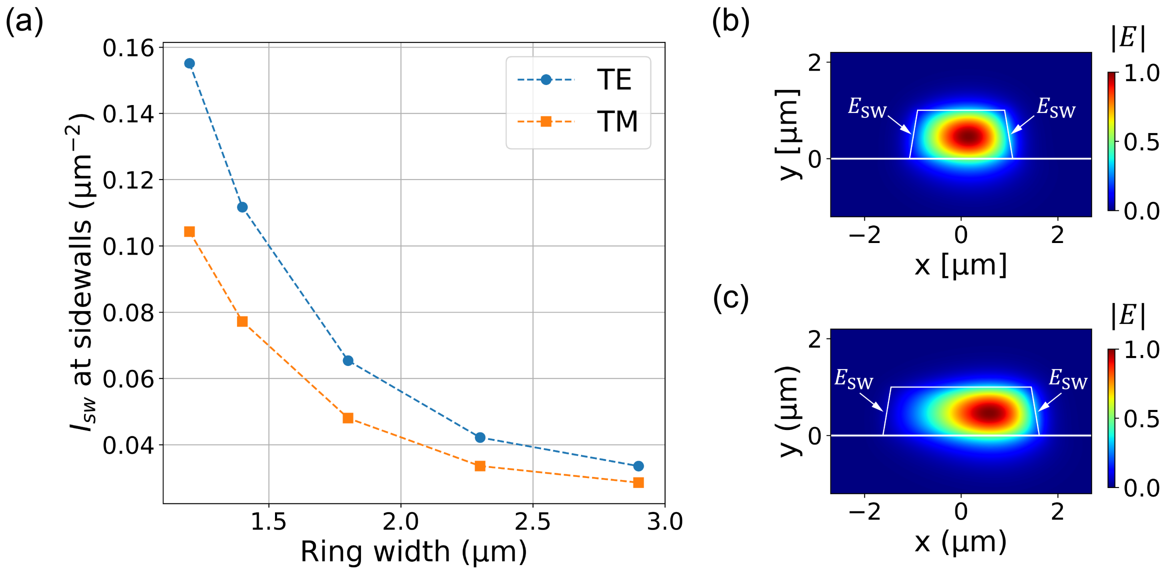

Figure S6: (a) Normalized field intensity I_{sw} at the sidewalls for MRRs of 60 μm radius and different ring widths. (b) and (c) Normalized TE_{00} field maps at λ =1550 nm for 1.8 μm- and 2.9 μm-wide MRRs, respectively, showing a reduced amount of field in the inner sidewall for the latter. The asymmetric mode shape is due to the radius of curvature of the MRRs.

In order to identify to which extent sidewall roughness is a limiting factor in the performance of our MRRs, we followed the same analytical approach as that described in Ref. 8. In optical WGs, sidewall-related scattering losses α_{sw} at fixed wavelength are proportional to:[9,10]

$$\alpha_{sw} \propto \frac{\sigma^2 (n_{core}^2 - n_{clad}^2)^2}{n_{eff}} \frac{\frac{1}{l}\int E_{sw}^2 \, dl}{\iint E^2 \, dx \, dy}, \tag{S2}$$

where σ is the sidewall roughness, n_{core} and n_{clad} are the refractive indices of the core and

cladding materials, respectively, and n_{eff} is the effective index of the mode. For the same level of surface roughness and approximating n_{eff} as a constant, we infer from Eq. (S2) that α_{sw} is proportional to the normalized average field intensity at the sidewalls $I_{\mathrm{sw}} \equiv (\frac{1}{l}\int E_{\mathrm{sw}}^2\,\mathrm{d}l)/\iint E^2\,\mathrm{d}x\,\mathrm{d}y$.

Through a series of finite-difference-eigenmode (FDE) simulations performed with Ansys Lumerical, we extracted the mode profile of all the MRRs studied in Figure 5 of the main text, and for each of them we calculated the normalized field intensity at the sidewall I_{sw}, for both the fundamental TE and TM modes. Results are shown in Figure S6(a), in which we can spot a decrease of I_{sw} with increasing ring width. This is due to the fact that wider ring wings experience a lower amount of sidewall field E_{sw}, as also noticeable from the mode profiles shown in Figures S6(b) and S6(c).

In particular, from Figure S6 we observe that for a ring width of 2.9 µm, I_{sw} is about half the value computed for a ring width of 1.8 µm. This means that, if propagation losses were only due to sidewall scattering, we should measure a Q_{int} value approximately twice larger for a ring width of 2.9 µm compared to 1.8 µm for both TE and TM polarizations. However, this is not in agreement with the experimental results obtained with sample B shown in Figure 5 of the main text, for which Q_{int} values for widths larger than 1.8 µm reach a plateau. This allows us to conclude that sidewall scattering is not the main factor limiting propagation losses for our wider MRRs. Intrinsic quality factors in our samples are therefore likely limited by other influences, such as the air pocket in the cladding at the bus-microring gap and the presence of PECVD-related O-H bonds in the cladding, as we also discuss in the main text.

S4 3D-FDTD simulation settings and convergence

In this section, we provide additional details on the finite-difference time-domain (FDTD) simulations of void-related propagation losses discussed in the main text. Simulations were performed with the three-dimensional (3D) FDTD module of Ansys Lumerical.[S1] We simulated light propagation in the telecom C-band of an AlN-on-sapphire WG (height: 1.1 µm, upper width: 1.4 µm) having voids. Voids were modeled as parallelepipeds with height H, in-plane diameter d, bottom coordinate y_{min} and areal density N. The definition of these quantities is also shown graphically on the refractive index map of Figure S7. The extent of the simulation region was set to 7 µm in width and 5.2 µm in height, and perfectly matched layer boundary conditions were adopted. The mesh refinement method was kept in the "conformal variant 0" default option, which is the most consistent scheme as advised by the Lumerical support service. The fundamental TE and TM modes were injected through input ports placed in a void-free region of the WG. The total WG region under the influence of voids was chosen to be 90 µm long, and frequency-domain power monitors were placed at a pitch of 10 µm, as shown in the top-view and lateral-view schemes of Figures S7(b) and S7(c). A fine simulation mesh

[S1] Ansys Lumerical FDTD v. 8.30.3536 from Lumerical Inc.

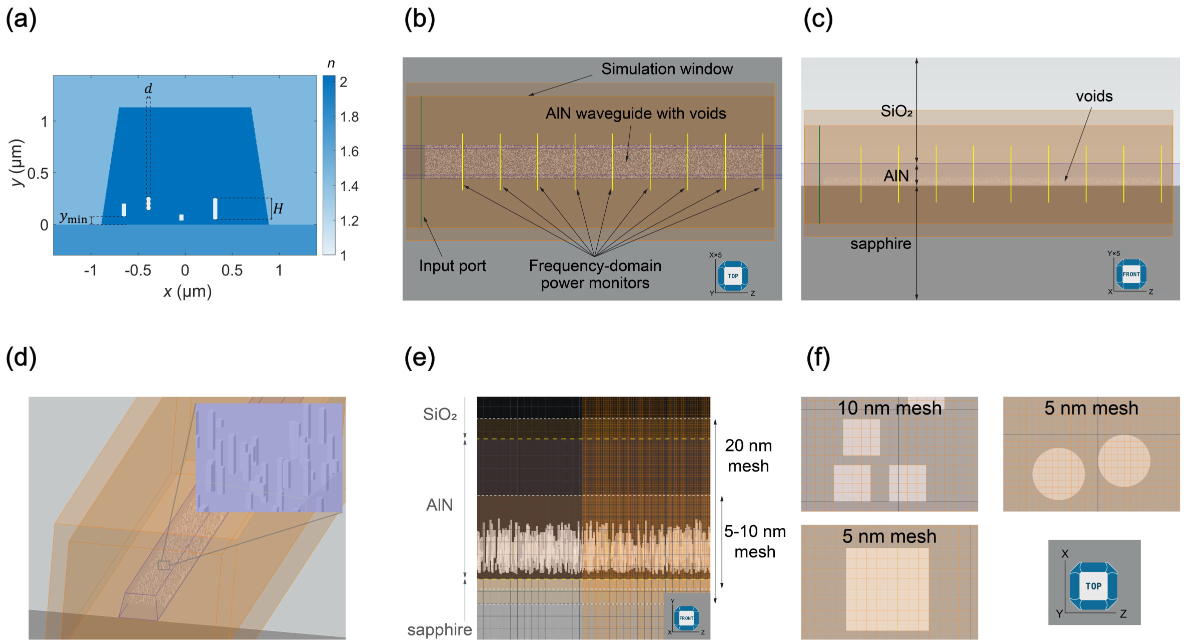

Figure S7: (a) Cross-section refractive index map of the simulated WG showing the presence of voids and the definition of relevant geometric parameters. (b) and (c) 3D-FDTD simulation setting schemes for the (x,z) and (y,z) planes, respectively. (d) 3D representation of parallelepiped-shaped voids inside a WG including a higher magnification inset. (e) (y,z) plane scheme showing the different meshes implemented in the simulations. (f) (x,z) meshing of voids with different geometries and mesh sizes.

of 10 nm was used in the bottom part of the WG, where voids are present. In the rest of the WG region—including at least 160 nm outside the WG—a 20 nm mesh was employed, while a coarser mesh was used in the remaining parts of the simulation domain. A lateral-view scheme picturing the various simulation meshes is shown in Figure S7(e).

Several tests were carried out to check the reliability and convergence of our simulation settings. The main results are reported in Table S1. For these tests, the following void parameters were adopted: H and y_{min} were randomly distributed and both lied in the 40-200 nm range while N and d were kept fixed at 100 μm^{-2} and 40 nm, respectively. A convergence test was performed by reducing the mesh size in the void region from 10 to 5 nm. In this latter case, the WG length under the influence of voids was shortened to 60 μm to reduce the computational load. Losses for both TE and TM polarizations were calculated using the transmission results from the power monitors with the method detailed in the main text. A comparison of the second and third rows of Table S1 shows that propagation losses vary by less than 5 % for the 10 and 5 nm meshes. Therefore, since computational times were about 5 times shorter with the 10 nm mesh, we adopted this configuration for all the simulations shown in the main text.

In addition, we also studied the effect of void geometry by simulating propagation losses for cylindrical voids with a diameter of 40 nm. In this case, a 5 nm mesh was adopted in order to better describe the round shape geometry of the voids. As can be seen from the last

Table S1: Convergence studies of 3D-FDTD simulation results consisting of analyzing optical losses for different mesh sizes. The effect of void shape is also taken into consideration.

Void shape	d	xz area (nm^2)	Mesh size (nm)	TE α ($dB\,cm^{-1}$)	TM α ($dB\,cm^{-1}$)
Parallelepiped	30	900	10	8.2 ± 0.1	5.1 ± 0.1
Parallelepiped	40	1600	10	21.7 ± 0.6	16.4 ± 0.3
Parallelepiped	40	1600	5	22.8 ± 0.8	16.8 ± 0.4
Cylinder	40	1257	5	15.0 ± 0.6	10.6 ± 0.3

row of Table S1, losses for cylindrical voids with $d = 40\,nm$ are in the range of values for parallelepiped voids with $d = 30$ and $40\,nm$. From this table, we can notice that the in-plane area of cylindrical voids with $d = 40\,nm$ ranges also between those of parallelepiped voids with $d = 30$ and $40\,nm$. This means that scattering is mainly dominated by the void size rather than by their exact in-plane geometry. The geometry still plays a role in the weight of propagation losses, but mainly in determining the ratio between TE and TM losses, as shown in the H sweep results reported in Figure 6(f) of the main text.

Finally, we comment on the choice of using frequency-domain power monitors to extract transmission results. Power monitors have the peculiarity of collecting all the optical power passing through the monitor region. This may be advantageous, as they also collect the power that is temporarily stored outside the fundamental propagating mode (e.g., in higher-order modes). However, they also capture part of the optical power scattered out of the WG that still propagates within the solid angle defined by the monitor. Therefore, we chose the monitor size in order to collect the optical power of the propagating mode while minimizing the collection of the scattered field. Nevertheless, some scattered light is still captured by the monitors, hence contributing to the loss calculations. By performing a longer simulation where we placed additional monitors after the WG region occupied by voids, we concluded that the values of the propagation losses extracted from the 3D-FDTD simulations are likely underestimated by approximately 8 %. Nonetheless, this remains the most reliable method we could find, given that monitoring only the optical power in the fundamental mode resulted in noticeable data oscillations, which made the loss estimation process more complicated and hence less reliable.

S5 Simulations of phase matching for SHG

In the main text, we show second-harmonic generation (SHG) from linear waveguides fabricated from sample C, pumped in the telecom range. To generate a second-harmonic (SH) signal, we targeted phase matching (PM) between the fundamental TM_{00} mode and the SH TM_{20} mode, as has been done in several previous studies on the same material platform.[11–13]

To support our experimental results, we report mode simulations of AlN WGs, performed with the FDE module of Ansys Lumerical. In Figure S8(a), we show the effective index (n_{eff}) dispersion of the two aforementioned modes in a WG with an upper width of 1.25 μm, deter-

mined by lithography, and a height of 1.14 µm, measured on the chip. Simulations show that varying the sidewall angle θ between 79° and 81°, within our measurement uncertainty, consistently yields PM in the telecom range, although the PM wavelength depends strongly on the exact value of θ. The experimentally observed peak position falls well within this uncertainty range, confirming a good agreement between simulations and experimental results. A similar behavior for the WG height (H) can be observed in Figure S8(b), which shows that variations in H within the experimental uncertainty, while keeping θ fixed at 80°, also cause non-negligible shifts in the PM wavelength.

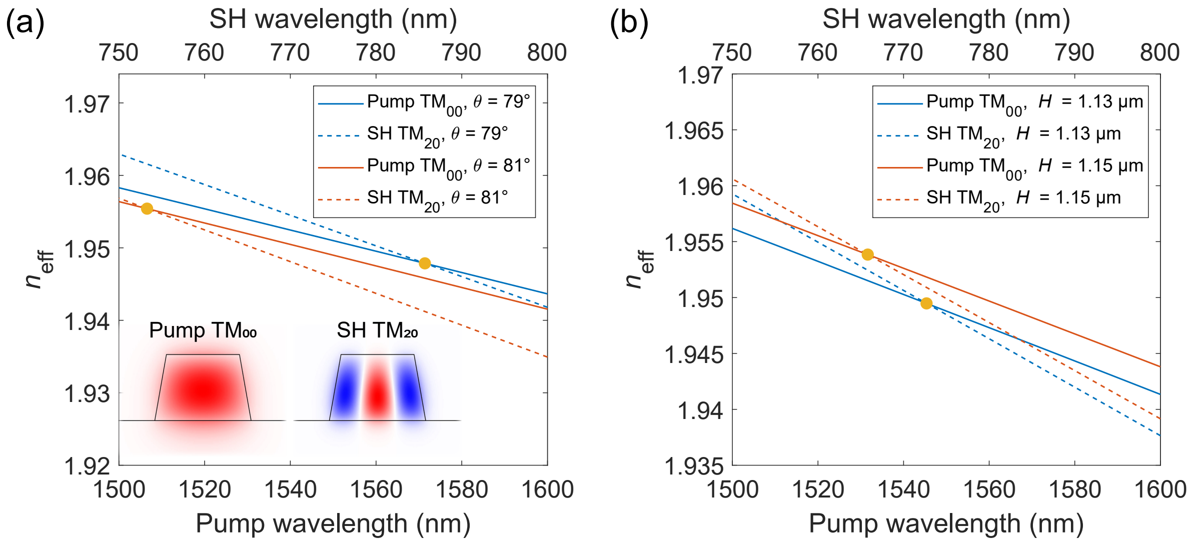

Figure S8: (a) Simulations of the effective index dispersion of the pump TM_{00} and the SH TM_{20} modes for a WG geometry of $1.25 \times 1.14\,\mu m^2$ and varying sidewall angles θ. The intersections between the two lines (yellow dots) correspond to the phase-matching conditions for the respective geometries. (b) Simulations for a WG width of 1.25 µm, a sidewall angle of 80°, and varying WG height H.

References

[1] Peters, L.; Meyer, T.; Margenfeld, C.; Spende, H.; Waag, A. Parasitic $Al_xO_yN_z$ surface defects on high-temperature annealed AlN and their role in hillock formation. *Appl. Phys. Lett.* **2023**, *123*, 112104.

[2] Pampili, P.; Pristovsek, M. Nitrogen-polar growth of AlN on vicinal (0001) sapphire by MOVPE. *J. Appl. Phys.* **2024**, *135*, 195303.

[3] He, C.; Zhao, W.; Wu, H.; Liu, N.; Zhang, S.; Li, J.; Jia, C.; Zhang, K.; He, L.; Chen, Z.; Shen, B. Fast growth of crack-free thick AlN film on sputtered AlN/sapphire by introducing high-density nano-voids. *J. Phys. D: Appl. Phys.* **2020**, *53*, 405303.

[4] Davydov, V. Y.; Kitaev, Y. E.; Goncharuk, I. N.; Smirnov, A. N.; Graul, J.; Semchinova, O.; Uffmann, D.; Smirnov, M. B.; Mirgorodsky, A. P.; Evarestov, R. A. Phonon dispersion and Raman scattering in hexagonal GaN and AlN. *Phys. Rev. B* **1998**, *58*, 12899–12907.

[5] Yang, S.; Miyagawa, R.; Miyake, H.; Hiramatsu, K.; Harima, H. Raman Scattering Spectroscopy of Residual Stresses in Epitaxial AlN Films. *Appl. Phys. Express* **2011**, *4*, 031001.

[6] Chang, C.; Abe, T.; Esashi, M. Trench filling characteristics of low stress TEOS/ozone oxide deposited by PECVD and SACVD. *Microsyst. Technol.* **2004**, *10*, 97–102.

[7] Sun, Y.; Shin, W.; Laleyan, D. A.; Wang, P.; Pandey, A.; Liu, X.; Wu, Y.; Soltani, M.; Mi, Z. Ultrahigh Q microring resonators using a single-crystal aluminum-nitride-on-sapphire platform. *Opt. Lett.* **2019**, *44*, 5679–5682.

[8] Liu, X.; Bruch, A. W.; Gong, Z.; Lu, J.; Surya, J. B.; Zhang, L.; Wang, J.; Yan, J.; Tang, H. X. Ultra-high-Q UV microring resonators based on a single-crystalline AlN platform. *Optica* **2018**, *5*, 1279–1282.

[9] Deri, R.; Kapon, E. Low-loss III-V semiconductor optical waveguides. *IEEE J. Quantum Electron.* **1991**, *27*, 626–640.

[10] Payne, F. P.; Lacey, J. P. R. A theoretical analysis of scattering loss from planar optical waveguides. *Opt. Quantum Electron.* **1994**, *26*, 977–986.

[11] Pernice, W. H.; Xiong, C.; Schuck, C.; Tang, H. X. Second harmonic generation in phase matched aluminum nitride waveguides and micro-ring resonators. *Appl. Phys. Lett.* **2012**, *100*, 223501.

[12] Guo, X.; Zou, C.-L.; Tang, H. X. Second-harmonic generation in aluminum nitride microrings with 2500%/W conversion efficiency. *Optica* **2016**, *3*, 1126–1131.

[13] Bruch, A. W.; Liu, X.; Guo, X.; Surya, J. B.; Gong, Z.; Zhang, L.; Wang, J.; Yan, J.; Tang, H. X. 17 000%/W second-harmonic conversion efficiency in single-crystalline aluminum nitride microresonators. *Appl. Phys. Lett.* **2018**, *113*, 131102.
